# Prevalence of Genes of *OXA-23* Carbapenemase and *AdeABC* Efflux Pump Associated with Multidrug Resistance of *Acinetobacter baumannii* Isolates in the ICU of a Comprehensive Hospital of Northwestern China

**DOI:** 10.3390/ijerph120810079

**Published:** 2015-08-21

**Authors:** Wei Jia, Caiyun Li, Haiyun Zhang, Gang Li, Xiaoming Liu, Jun Wei

**Affiliations:** 1 Ningxia Key laboratory of Clinical and Pathogenic Microbiology, Yinchuan, Ningxia 750004, China; E-Mails: jiawei6365@126.com (W.J.); lcy77720150427777@163.com (C.L.); gone.lee@163.com (G.L.); liuxiaoming@nxmu.edu.cn (X.L.); 2 Center of Laboratory Medicine, the General Hospital of Ningxia Medical University, Yinchuan, Ningxia 750004, China; E-Mail: zhy34@163.com (H.Z.); 3 The Graduate school, Ningxia Medical University, Yinchuan, Ningxia 750004, China; 4 The First People’s Hospital of Mudanjiang City, Mudanjiang, Heilongjiang 157011, China

**Keywords:** *Acinetobacter baumannii*, multidrug resistance, nosocomial infections, oxacillinase, efflux pump

## Abstract

The objective of this study was to explore the molecular epidemiology and the genetic support of clinical multidrug resistant (MDR) *Acinetobacter baumannii* (*A. baumannii*) isolates in an ICU ward of a comprehensive hospital. A total of 102 non-duplicate drug-resistant *A. baumannii* isolates were identified and 93 (91.1%) of them were MDR strains. Molecular analysis demonstrated that carbapenemase genes *bla*_OXA-23_ and *bla*_OXA-51_ were presented in all 93 MDR isolates (100%), but other carbapenemase genes, including *bla*_OXA-24_, *bla*_OXA-58_, *bla*_IMP-1_, *bla*_IMP-4_, *bla*_SIM_, and *bla*_VIM_ genes were completely absent in all isolates. In addition, genes of *AdeABC* efflux system were detected in 88.2% (90/102) isolates. Interestingly, an addition to efflux pump inhibitor, reserpine could significantly enhance the susceptibility of MDR isolates to moxifloxacin, cefotaxime, and imipenem (*p* < 0.01). Clonal relationship analysis further grouped these clinical drug-resistant isolates into nine clusters, and the MDR strains were mainly in clusters A, B, C, and D, which include 16, 13, 25, and 15 isolates, respectively. This study demonstrated that clinical isolates carrying carbapenemase-encoding genes *bla*_OXA-23_ and *AdeABC* efflux pump genes are the main prevalent MDR *A. baumannii*, and the co-expression of oxacillinase and efflux pump proteins are thus considered to be the important reason for the prevalence of this organism in the ICU of this hospital.

## 1. Introduction

The emergence of multidrug-resistant (MDR) bacterial strains has been recognized as a main challenge for treatment of clinical infection with broad-spectrum antibiotics. *Acinetobacter baumannii* (*A. baumannii*) is an emerging opportunistic nosocomial pathogen with great concern worldwide, which is the most common clinically isolated *Acinetobacter* species and a cause of severe infections in intensive care units (ICU) of hospitals, owing to its remarkable ability to acquire resistance to most antimicrobials [1,2]. Carbapenem, aminoglycosides, and quinolone antibiotics are often efficient in the treatment of *A. baumannii* infection. However, MDR *A. baumannii* isolates have recently been increasingly reported in many countries, particularly in Asia-Pacific countries including China [3,4,5,6,7,8,9,10,11,12]. A recent surveillance report from CHINET for the resistance rates of *Acinetobacter* species shows that *A. baumannii* isolates accounted for 89.6% of the resistance, and their resistances to imipenem and meropenem are up to 62.8% and 59.4% in China, respectively (http://narin.minke.cn) [12].

The molecular basis of multidrug resistance in this species has been attributed to combined mechanisms of an increased expression of oxacillinase (OXA)-type carbapenemases and non-enzymatic mechanisms, such as increased cell membrane impermeability, expression of multidrug efflux pump proteins, and/or and alterations in penicillin-binding proteins, due to a high level of genomic plasticity and mutation of endogenous genes associated with antimicrobial resistance [13,14,15,16]. An analysis of the molecular epidemiology of nosocomial infection MDR *A. baumannii*, particularly in the ICU ward of a hospital thus may help to develop efficient guidelines to control the spread of these bugs [17].

To date, there are only few reports demonstrating combinations of different mechanisms of resistance in MDR bacterial pathogens, despite this there is an apparent correlation of antimicrobial resistance with the carbapenemase production and an expression of multidrug efflux pumps in *A. baumannii* has been reported [18,19,20,21]. In the present study, we interrogated the molecular mechanism with a focus in the genetic linkage of OXA-type carbapenemases and multidrug efflux pumps in drug-resistant *A. baumannii* isolates recovered from the ICU ward in the General Hospital of Ningxia Medical University, a national comprehensive hospital in Northwestern China from January 2013 to July 2014. 

## 2. Experimental Section 

### 2.1. Bacterial Isolation 

The Clinical Research Ethics Committee at the General Hospital of Ningxia Medical University approved this study with a waiver for informed consents. All of the non-duplicate clinical isolates were routinely collected from patients who were not received an antibiotic therapy at the General Hospital of Ningxia Medical University (Yinchuan, China) in the ICU from January 2013 to July 2014. Isolated bacteria were stored at −80 °C prior to be used in this study. All clinical strains of *Acinetobacter* spp. were identified with a ViteK-2 Compact automated microbiological system (Biomerieux, France). The Microseq 500 16S rDNA bacterial identification kit was used to identify isolates for *Acinetobacter* species (Applied Biosystem, Foster City, CA, USA).

### 2.2. Test of Antimicrobial Susceptibility 

The susceptibility of an antimicrobial agent was determined using E-test strips (AB Biodisk, Slona, Sweden) per manufacturer’s instruction. The susceptibility was interpreted according to the guidelines of the Clinical Laboratory Standards Institute (CLSI) [22,23], and defined as previously described [24]. *Escherichia coli* ATCC 25922 and *Pseudomonas aeruginosa* ATCC 27853 strains were used as references for antimicrobial susceptibility testing. A total of 102 drug-resistant *A. baumannii* isolates were recovered from patients hospitalized in the ICU ward of this hospital, among which 93 were multi-drug resistant strains and 9 were resistant to less than three classes of the following 11 tested antibiotics or a synergistic combination. The tested antibiotics in this study were ampicillin-sulbactam, cefepime, ceftazidime, ceftriaxome, imipenem, levofloxacin, piperacillin, polymyxin B, ticarcillin/clavulanicac, tobramycin and trimethoprim/sulfamethoazole. A multidrug resistant *A. baumannii* was defined as an isolate resistant to at least three classes of antibiotics, and isolates resistant to less than three classes of antibiotics were designated as antibiotic unsusceptible isolates in this study. In order to examine the effect of efflux pumps in antimicrobial resistance, the MICs of cefotaxime, moxifloxacin and imipenem for *A. baumannii* were measured in the presence of an efflux pump inhibitor (EPI) reserpine (Dalian Meilun Biology Technology Co., Ltd., Dalian, China) at a concentration of 25 mg/L with an agar dilution method as described previously [25]

### 2.3. Identification of the Drug Resistance Genes

All isolates were subjected to detect genes of drug resistance, including the carbapenem-resistance genes (*bla_NDM-1_*, *bla_SIM_*, *bla_VIM_*, *bla_IMP-1_*, *bla_IMP-4_*, *bla_OXA-23_*, *bla_OXA-24_*, *bla_OXA-58_*, *bla_OXA-51_*) and the efflux pump genes *(AdeA*, *AdeB*, *AdeC*, *AdeR*, *AdeS*) by a polymerase chain reaction (PCR) assay as described elsewhere [26]. All primers used for PCR in this study were listed in the Table 1. Genomic DNA of *A. baumannii* isolates was extracted using TIANamp Bacteria DNA Kit (Tiangen, Beijing, China). PCR was performed using Taq PCR Master Mix (TaKaRa, Dalian, China). The cycling parameters of PCR were as follows: an initial denaturation at 94 °C for 5 minutes, followed by 30 cycles of 94 °C denature for 30 seconds, 55 °C annealing for 30 seconds and 72 °C extension for 90 seconds. Then the PCR products were resolved in Ethidium Bromide (EM) agarose gels and visualized under an Ultraviolet (UV) light.

**Table 1:** Sequences of PCR primer sets for genes of carbapenemase and ABC efflux pumps of A. baumannii used in this study.

Target genes	Primer sets	Primer sequence (5′→3′)	Amplicon Size (bp)
*Carbapenemase*	*bla*_NDM-1_ F	GCATTGGCGGCGAAAGTCA	921
	*bla*_NDM-1_ R	CTCGCACCGAATGTCTGGC	
	*bla*_SIM_ F	TACAAGGGATTCGGCATCG	741
	*bla*_SIM_ R	TAATGGCCTGTTCCCATGTG	
	*bla*_VIM_ F	TTATGGAGCAACCGATGT	920
	*bla*_VIM_ *R*	CAAAAGTCCCGCTCCAACGA	
	*bla*_IMP-1_ F	ATCCAAGCAGCAAGCGCGTTA	474
	*bla*_IMP-1_ R	AGGCGTGCTGCTGCAACGACTTGT	
	*bla*_IMP-4_ F	CTACCGCAGCAGAGTCTTTG	879
	*bla*_IMP-4_ R	AACCAGTTTTGCCTTACCAT	
	*bla*_OXA-23_ F	GATGTGTCATAGTATTCGTCG	774
	*bla*_OXA-23_ R	TCACAACAACTAAAAGCACTG	
	*bla*_OXA-24_ F	TTCCCCTAACATGAATTTGT	828
	*bla*_OXA-24_ R	GTACTAATCAAAGTTGTGAA	
	*bla*_OXA-58_ F	AAGTATTGGGGCTTGTGCTG	800
	*bla*_OXA-58_ R	CCCCTCTGCGCTCTACATAC	
	*bla*_OXA-51_ F	TAATGCTTTGATCGGCCTTG	760
	*bla*_OXA-51_ R	TGGATTGCACTTCATCTTGG	
*Multidrug resistance efflux pumps*	*AdeA* F	GGCGTATTGGGCAATCTTTTGT	1157
	*AdeA* R	GTCACCGACTTTCAAGCCTTTG	
	*AdeB* F	TGGCGGAATGAAGTATGT	1323
	*AdeB* R	GCAGTGCGGCAGGTTAG	
	*AdeC* F	GACAATCGTATCTCGTGGACTC	1331
	*AdeC* R	AGCAATTTTCTGGTCAGTTTCC	
	*AdeR* F	TCACATGGCTATCTACGGTTGG	538
	*AdeR* R	TGAAGGCATGAGTGTTATTCGG	
	*AdeS* F	GTGGACGTTAGGTCAAGTTCTG	949
	*AdeS* R	TGTTATCTTTTGCGGCTGTATT	

### 2.4. Genetic Relationship among A. Baumannii Isolates Determined by Pulse Field Gel Electrophoresis (PFGE) 

Pulsed-field gel electrophoresis (PFGE) was performed as previously described [27]. Briefly, the purified bacterial genomic DNA was digested by the restriction enzyme ApaI (TaKaRa, Dalain, China), and the digested product was separated in a Bio-Rad CHEF-Mapper apparatus with parameters of pulses ranging from 5 to 20 seconds at a voltage of 5 V/cm and switch angle of 120° at 14 °C for 19 h (Bio-Rad Laboratories, Hercules, CA, USA). The gel was then stained with ethidium bromide and the restricted pattern of DNAs was acquired using Bio-Rad Vilber Lourmat. The BioNumerics 6.6 software (Applied Maths, Kortrijk, Belgium) was employed for analyzing the similarities between the digitized PFGE profiles. The similarity between DNA restriction profiles were interpreted according to the criteria described by Seifert *et al.* [28] using the Dice correlation coefficient F. F = 2Nxy/(Nx + Ny), where Nxy represents the number of identical bands between isolate x and y, and the Nx and Ny are total numbers of bands acquired from the digestion in isolates x and y, respectively. Isolates with a similarity of >85% following dendrogram analysis were considered to represent an identical PFGE genotype (pulsotype) and categorized into the same group [28].

### 2.5. Statistical Analysis

All data were managed using the WHONET version 5.6 software. The statistical analysis was processed with the Statistical Package for the Social Sciences (SPSS) software version 18.0 (SPSS, Chicago, IL, USA). The changes of MICs for MDR *A. baumanniican* between the presence and absence of reserpine were compared with a t-test analysis. A *p* < 0.05 was defined as a statistical significance.

## 3. Results 

### 3.1. Epidemiological Data of Drug-Resistant A. Baumannii Infection in ICU 

During the study period, a total of 102 non-duplicated drug-resistant *A. baumannii* isolates to tested antibiotics were collected from the ICU ward of this hospital. The tested antibiotics or synergistic combinations in this study include ampicillin-sulbactam, cefepime, ceftazidime, ceftriaxome, imipenem, levofloxacin, piperacillin, polymyxin B, ticarcillin/clavulanicac, tobramycin, and trimethoprim/sulfamethoazole. The age of patients ranged from 1 to 92 years (median, 55 years); 78 (76.47%) patients were males and 24 (23.53%) were females. The majority of drug-resistant strains were recovered from respiratory specimens (72/102), followed by samples from body secretions (9/102), bloodstream (6/102), urine (5/102), chest drainage fluid (4/102), pus (4/102), and cerebrospinal fluid (2/102). Among the 102 drug-resistant isolates, 93 out of the 102 isolates were categorized as MDR *A. baumannii* which were resistant to at least three classes of antimicrobials; nine of them were identified as drug-resistance to less than three classes of the tested antibiotics, which were designated as antibiotic unsusceptible isolates in this study (Table 2). All of the 102 drug-resistant clinical isolates were resistant to trimethoprim/sulfamethoazole (102/102, 100%); high resistant rates were also observed to cefepime (94/102, 92.2%), ceftazidime (94/102, 92.2%), ceftriaxome (99/102, 97.1%), imipenem (83/102, 81.4%), levofloxacin 92/102, 90.2%), piperacillin (99/102, 97.1%), ticarcillin/clavulanicac (98/102, 96.1%), and tobramycin (90/102, 88.2%) (Table 2). Noteworthy, in spite of 13 out of the 102 (12.7%) drug-resistant clinical isolates were resistant to polymyxin B, *A. baumannii* isolates showed most susceptible to this drug of “last resort” in this study [6] (Table 2). 

**Table 2:** The susceptibilities of clinical *A. baumannii* isolates to tested antibiotics (N = 102).

Antibiotics	Drug-resistant isolates	MIC ranges (mg/mL)	Drug-susceptible isolates
Trimethoprim/sulfamethoazole	102/102, (100%)	4–32/64	0/102, (0.0%)
Piperacillin	99/102, (97.1%)	128–512	3/102, (2.9%)
Ceftriaxome	99/102, (97.1%)	16–256	3/102, (2.9%)
Ampicillin-sulbactam	98/102, (96.1%)	8–256/8	4/102, (3.9%)
Ticarcillin-clavulanicac	98/102, (96.1%)	32–1224/1	4/102, (3.9%)
Cefepime	94/102, (92.2%)	8–512	8/102, (7.8%)
Ceftazidime	94/102, (92.2%)	4–128	8/102, (7.8%)
Levofloxacin	92/102, (90.2%)	2–64	10/102, (9.8%)
Tobramycin	90/102, (88.2%)	4–256	12/102, (9.8%)
Imipenem	83/102, (81.4%)	2–64	19/102, (18.6%)
Polymyxin B	13/102, (12.7%)	1–16	89/102, (87.3%)

### 3.2. Genes of Antimicrobial Resistance Identified by Multiplex PCR Assays 

To uncover the underlying mechanism involved in the drug-resistance of these 102 *A. baumannii* isolates, genes encoding carbapenemase and efflux pumps were determined by multiplex PCR assays. The distribution of carbapenemase and efflux pump genotypes in drug-resistant strains was shown in Table 3. There were no carbapenemase genes *bla*_OXA-23_, *bla*_OXA-24_, *bla*_OXA-51_, *bla*_OXA-58_, *bla*_VIM_, *bla*_IMP-1_, *bla*_IMP-4_, *bla*_SIM_, *bla*_NDM-1_ detected in all nine antibiotic unsusceptible isolates, while efflux pump *AdeA*, *AdeB*, *AdeC* genes and their regulator *AdeR* and *AdeS* genes were found in these isolate (data not shown). Of note, *bla*_OXA-23_, *bla*_OXA-51_ genes were present in all 93 MDR *A. baumannii* isolates, while the other carbapenemase genes, *bla*_oxa-24_, *bla*_OXA-58_, *bla*_VIM_, *bla*_IMP-1_, *bla*_IMP-4_, *bla*_SIM_, *bla*_NDM-1_ were completely absent (Table 3). Among efflux genes, the majority of the MDR isolates were found to harbor *AdeABC* efflux pump genes, and only 11.8% (12/102) of the isolates were undetectable for *AdeABC* and *AdeRS* genes (Table 3). The *AdeA*, *AdeB*, *AdeC*, *AdeR* and *AdeS* genes were detected in 79.6% (74/93), 77.4% (72/93), 86.0% (80/93), 81.7% (76/93), and 80.6% (75/93) of the 93 MDR isolates, respectively. Equally noteworthy, there were 73 and 68 out of the 93 MDR isolates harbored *AdeABC* and *AdeABCRS* multiple genes, respectively. The *AdeA*, *AdeB*, *AdeC*, *AdeR*, and *AdeS* genes were detected in 4/9, 1/9, 3/9, 2/9, and 1/9 of the 9 of the antibiotic unsusceptible isolates, respectively (Table 3).

**Table 3:** Distribution of *Carbapenemase* genes and *Ade* efflux pump genes in the 102 clinical drug-resistant *A. baumannii* isolates in this study (Number of isolates harboring *Carbapenemase* gene/number of isolates containing efflux pump gene).

Genes	*AdeA*	*AdeB*	*AdeC*	*AdeS*	*AdeR*
*bla*_OXA-23_	93/78	93/73	93/83	93/78	93/76
*bla*_OXA-24_	0/78	0/73	0/83	0/78	0/76
*bla*_OXA-51_	93/78	93/73	93/83	93/78	93/76
*bla*_OXA-58_	0/78	0/73	0/83	0/78	0/76
*BlaNDM-1*	0/78	0/73	0/83	0/78	0/76
*BlaSIM*	0/78	0/73	0/83	0/78	0/76
*Bla*_VIM_	0/78	0/73	0/83	0/78	0/76
*Bla*_IMP-1_	0/78	0/73	0/83	0/78	0/76
*Bla*_IMP-4_	0/78	0/73	0/83	0/78	0/76

### 3.3. Impact of Efflux Pumps on the Susceptibility of Clinical MDR A. Baumannii Isolates to Antibiotics

**Figure 1:**
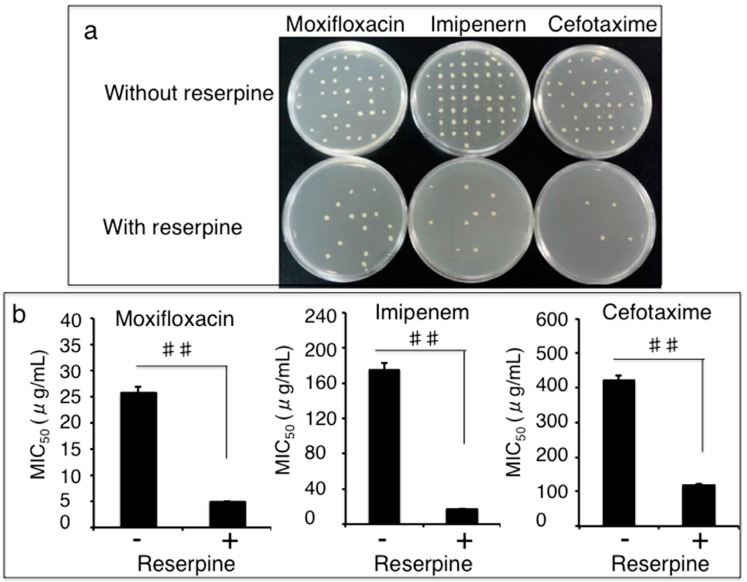
Impact of reserpine on the susceptibility of MDR *A. baumannii* to antibiotics. The clinical drug-resistant *A. baumannii* isolates were culture in the presence of cefotaxime, moxifloxacin, or imipenem with or without 25 mg/L of efflux pump inhibitor reserpine, the MICs were determined by agar dilution method. (**a**) Representative images showed an enhanced inhibitory activity of indicated antibiotics in the presence of reserpine. (**b**) Effect of reserpine on the susceptibility of MDR *A. baumannii* to antibiotics. An addition of reserpine resulted in a significantly reduction of MICs of indicated antibiotics the clinical isolates (*p* < 0.01), indicative of an enhanced susceptibility to these antimicrobials. Compared to the corresponding reserpine absent group, ##: *p* < 0.01. Data represented the mean ± SD from three independent triplicated experiments (N = 102).

We next sought to explore whether the efflux pumps played a role in the drug-resistance of these 102 clinical isolates of *A. baumannii* to antibiotics by an active efflux inhibition test on the M-H agar plates with or without 25 mg/L of efflux pump inhibitor (EPI) reserpine. The presence of reserpine showed a dramatically enhanced inhibitory capacity of moxifloxacin, cefotaxime, and imipenem to the growth of the clinical MDR *A. baumannii* isolates (Figure 1a). In the presence of reserpine, the susceptibilities of 80 out of the 93 MDR isolates (86.0%, 80/93) to moxifloxacin, 75 of the 93 isolates to cefotaxime (80.6%, 75/93), and 88 of the 93 MDR isolates (94.6%, 88/93) to imipenem were increased by 2–8, 2–4, and 2–32 folds as determined by an MIC assay, respectively (Figure 1b). The reserpine-mediated decreases of MICs of these MDR isolates were statistically different in comparison with they were treated with the antibiotics alone (*p* < 0.01) (Figure 1b), indicating that a bacterial antibiotic efflux mechanism was involved in the clinical MDR isolates. Intriguingly, the presence of reserpine had no inhibitory effect on the growth and MICs of the nine antibiotic unsusceptible *A. baumannii* isolates (data not shown).

### 3.4. Clonal Relationship of Drug-Resistant A. Baumannii Isolates Determined by a Pulsed-Field Gel Electrophoresis (PFGE) Method 

In order to identify clonal relationship of the 102 clinical drug-resistant *A. baumannii* isolates, PFGE analysis was employed using Apa I-digested *A. baumannii* genomic DNA. The 102 clinical isolates gave 47 reproducible ApaI-digested DNA profiles (PFGE genotypes) with a Dice coefficient, F ranging from 0.85 to 1.00 [28]. Cluster analysis of the pulsotypes grouped the 102 clinical drug-resistant *A. baumannii* isolates into nine clusters with a cutoff point at 85% similarity (Table 4). The MDR isolates were grouped into four main clusters, A, B, C, and D, which had respective 16, 13, 25 and 15 strains, while none of the nine antibiotic unsusceptible isolates was in the clusters A–D. Interestingly, isolates in cluster B and E exhibited a tendency of *AdeABC* efflux pump genotypes (Table 4). This result suggested that the majority of the MDR isolates had the diversity with multivariate clones. 

**Table 4:** Distribution of *AdeABC* efflux pump genes in different groups of clinical drug-resistant *A. baumannii* isolates.

Efflux pump *AdeABC* genes	PFGE groups						Constituent ratio
	A	B	C	D	E	Other	
*AdeABC*, *AdeRS*	11	12	18	5	3	19	68/102 (66.7%)
*AdeABC*	0	1	0	0	0	3	4/102 (3.9%)
*AdeABC*, *AdeR*	0	0	0	0	0	2	2/102 (1.9%)
*AdeABC*, *AdeS*	2	0	2	0	0	0	4/102 (3.9%)
Other genotypes	3	0	1	3	0	5	12/102 (11.8%)
Negative	0	0	4	7	0	1	12/102 (11.7%)
Sum	16	13	25	15	3	30	102/102 (100%)

## 4. Discussion

An increasing emergence of antibiotic resistance in microbes has had significant impact on the patient outcomes and challenges treatments of clinical infection using broad-spectrum antibiotics. Moreover, *A. baumannii* recently emerged as an important pathogen responsible for epidemics of nosocomial infections, particularly in the ICU ward of a hospital [1]. Therefore, an identification of molecular mechanisms of drug resistance in *A. baumannii* will improve treatments of hospital-acquired infections and help for developing appropriate control measures to prevent further spread of multidrug-resistant organism [10]. In the present study, we investigated possible molecular epidemic mechanisms of MDR *A. baumannii* in the ICU ward of the General Hospital of Ningxia Medical University in Northwestern China, and found that most common MDR *A. baumannii* strains identified in the ICU of this hospital were isolates harboring genes of class D oxacillinases *bla*_OXA-23_/*bla*_OXA-51_ and drug-resistant efflux pump *AdeABC* in the period of January 2013 to July 2014. Total of 102 clinical drug-resistant *A. baumannii* isolates were recovered, and PFGE analysis further revealed that the MDR isolates were mainly grouped into A, B, C, and D clusters. Furthermore, these drug-resistant isolates displayed a relative low resistance to polymyxin B (12.5%) but high resistance to trimethoprim/sulfamethoazole (100%); an addition of efflux pump inhibitor reserpine could significantly enhance the susceptibility of these MDR *A. baumannii* strains to moxifloxacin, cefotaxime, and imipenem. 

Mechanisms of MDR *A. baumannii* are complex. In addition to a remarkable ability of this organism to horizontally acquire resistance determinants, intrinsic resistance mechanisms include production of enzyme, change of outer membrane permeability, expression of drug resistance and efflux pump genes [15]. For instance, *A. baumannii* is able to gain its resistance to carbapenems mainly through a mechanism of producing different carbapenemase enzymes including class B metallo-b-lactamases (MBLs) and class D oxacillinases (OXAs) [29]. *bla*_OXA-23_, *bla*_OXA-24_, *bla*_OXA-51_ and *bla*_OXA-58_ are most common class D *bla*_OXAs_ reported in clinical MDR *A. baumannii* isolates, particularly in Asia-Pacific countries [4,9,30,31], where MDR *A. baumannii* isolates harboring *bla*_OXA-23_ gene were more prevalent than any other gene type, and the *bla*_OXA-58_ gene was rarely detected in these strains [31]; while MBLs IMP, VIM, and SIM-producing *A. baumannii* isolates have also been often reported worldwide [29]. 

In line with the findings from other studies of Asia-Pacific countries including China and Korea [4,8,9,10,30,31,32], the majority of prevalent MDR *A. baumannii* in the ICU of this hospital were strains carrying *bla*_OXA-23_ and *bla*_OXA-51_ genes, of which were detected in all of the 93 MDR *A. baumannii* isolates, but none of the *bla*_OXA-24_, *bla*_OXA-58_, *bla*_VIM_, *bla*_IMP-1_, *bla*_IMP-4_, *bla*_SIM_, *bla*_NDM-1_ genes were detected in these drug-resistant isolates. Of note, since *bla*_OXA-51_ gene is an intrinsic, chromosomal carbapenemase naturally present in all *A. baumannii* strains regardless of drug susceptibility, it is an intrinsic cambapenem resistance mechanism. Therefore, it has been used as a target gene for identification of *A. baumannii species* using PCR, which was correlated well with 16S rRNA and *rpoB* sequencing [33]. Interestingly, all the 93 MDR isolates were *A. baumannii* strains, but the nine antibiotic unsusceptible isolates lacking *bla*_OXA-51_ gene might be *Acinetobacter* spp. strains. Such a high frequency of *bla*_OXA-51_ gene detected in these MDR isolates may imply that an intrinsic drug resistance mechanism also contribute the multidrug resistance. For instance, the porin deficiency is another intrinsic carbapenem resistance mechanism in *A. baumannii* [15]. Porins are outer membrane proteins (OMPs) able to form transport channels for molecules crossing membranes. The carbapenem-associated OMP (CarO) is the most characterized porin and the best characterized causes of intrinsic carbapenem resistance in *A. baumannii* [15,34]. An alteration of CarO gene expression could contribute imipenem resistance by reducing the penetration of drug into the cells [15,34,35].

In terms of the genetic basis of *bla*_OXA-23_ dissemination in *A. baumannii* in China, Liu *et al.* recently uncovered that the plasmid pAZJ221 and Tn2009 might effectively contribute the broad dissemination of *bla*_OXA-23_ gene in *Acinetobacter* spp. in China, suggesting that the mechanism of horizontal gene transfer may play a key role in the dissemination of *bla*_OXA-23_ gene in this country [30]. Of interest, despite a remarkably increased proportion of MDR *A. baumannii* isolates carrying the *bla*_OXA-23_ gene has been reported in Asia-Pacific region since last decade, while the prevalence of MDR *A. baumannii* harboring *bla*_OXA-51_ gene has been decreased [4,10,31]. Differing from these observations, the *bla*_OXA-51_ gene was determined in all of the 93 MDR *A. baumannii* isolates in this study. However, the contribution of *bla*_OXA-51_ gene in the MDR of these isolates needs to be further identified by a quantitative assay. Together with other findings, this finding thus further supports a notion of that the prevalent *bla*_OXA_ genes are significantly varied depending on the time, place, and even hospital ward of isolation [36,37,38].

Apart from the production of carbapenemase, MDR efflux pumps also have displayed multifactorial roles in the resistance of *Acinetobacter* spp to antibiotics [15,19,39,40,41,42]. There are three resistance-nodulation-cell division (RND) systems, AdeFGH, AdeIJK, and AdeABC have been characterized in *A. baumannii*, among which the AdeABC was the most frequently involved in MDR *A. baumannii*, which was found in approximately 80% of clinical isolates [43]. *A. baumannii* overexpressing AdeABC has been reported to be significantly correlated with resistance to tigecycline, minocycline, and gentamicin and other biological functions [15]. 

Using a set of isogenic mutants of *A. baumannii* strains, Yoon *et al.* recently demonstrated that the expression of RND-efflux systems, particularly the AdeABC contributed to the drug resistance and biofilm formation in *A. baumannii* [44]. An *A. baumannii* mutant overproduced AdeABC could confer a clinical resistance to aminoglycosides. More importantly, the AdeABC pump showed a synergistic effect of the level of resistance of the host when it was in combination with enzymatic resistance to carbapenems and aminoglycosides, indicative of a synergistic effect between the expression of an efflux pump and a resistance gene on MDR of *A. baumannii* strains [44]. In this study, *AdeABC* genes were detected in the majority of clinical MDR isolates of those also harboring *bla*_OXA-23_ gene, and an addition of EPI reserpine led to an enhanced susceptibility of MDR isolates to antibiotics (*p* < 0.01). *A. baumannii* isolates carrying both of oxacillinase and efflux pump genes were also reported in several previous studies [19,41]. For instance, the expression both of *AdeABC* efflux pump and *bla*_OXA-23_ played a role in acquiring carbapenem resistant *A. baumannii* isolates in a hospital of Korea [41]. In addition, results from a study in imipenem resistant *A. baumannii* harboring *bla*_OXA-66_*/bla*_OXA-51_ genes by Hu *et al.* suggested that the production of carbapenemase could account for the intrinsic resistance to imipenem, but drug export by an efflux pump might contribute more in the prevalence of imipenem-resistant *A. baumannii* [19]. These studies and ours clearly suggest that the RND-type efflux systems and oxacillinase may play a synergistic role in multidrug resistance of *A. baumannii* in this hospital. 

## 5. Conclusions 

Collectively, 102 drug-resistant *A. baumannii* isolates were identified from the ICU ward of the General Hospital of Ningxia Medical University, China. The majority (93/102) of these isolates were MDR strains. Genotyping analysis revealed that 100% (93/93) of the MDR isolates carried carbapenemase genes *bla*_OXA-23_/*bla*_OXA-51_ but were absent other carbapenemase genes *bla*_OXA-24_, *bla*_OXA-58_, *bla*_VIM_, *bla*_IMP-1_, *bla*_IMP-4_, *bla*_SIM_, *bla*_NDM-1_. Importantly, most of the MDR *A. baumannii* isolates also carried genes of *AdeABC* efflux pumps, and a presence of efflux pump inhibitor reserpine could significantly enhance their susceptibility to antibiotics (*p* < 0.01). These clinical drug-resistant isolates could be grouped into nine clusters as determined by a PFGE analysis, and the MDR were mainly in clusters A, B, C, and D. These findings illustrate a challenge of increasing MDR *A. baumannii* isolates in the ICU ward. The high distribution of multiple genes, mainly the genes of *bla*_OXA-23_/*bla*_OXA-51_ carbapenemase and RND *AdeABC* efflux pump contributes to distinct drug-resistant mechanisms, which also indicates an emerging threat in this hospital. Therefore, local molecular detection of genes accounting for drug resistance in a hospital ward, such as the ICU is essential to limit the spread of nosocomial infections caused MDR *A. baumannii*. This result thus may be useful for developing an effective guidance to prevent nosocomial infections caused by *A. baumannii* in this hospital.

## List of abbreviations

*A. baumannii*: *Acinetobacter baumannii*
Ade: Acinetobacter drug efflux
ICU: Intensive care unit
IMP: metallo-β-lactamase resistant to imipenem
MDR: multidrug-resistant
NDM: New Delhi metallo-β-lactamase
MIC: Minimal inhibitory concentration
OXA: oxacillinase
PCR: polymerase chain reaction
PFGE: pulsed-field gel electrophoresis
SIM: seoul imipeneamase
VIM: verona integron encoded metallo-β-lactamase
